# Prediction of BAP1 Expression in Uveal Melanoma Using Densely-Connected Deep Classification Networks

**DOI:** 10.3390/cancers11101579

**Published:** 2019-10-16

**Authors:** Muyi Sun, Wei Zhou, Xingqun Qi, Guanhong Zhang, Leonard Girnita, Stefan Seregard, Hans E. Grossniklaus, Zeyi Yao, Xiaoguang Zhou, Gustav Stålhammar

**Affiliations:** 1 School of Automation, Beijing University of Posts and Telecommunications, Beijing 100876, China; sunmuyi@bupt.edu.cn (M.S.); XingqunQi@bupt.edu.cn (X.Q.); zghzgh1779@bupt.edu.cn (G.Z.); yaozeyi@bupt.edu.cn (Z.Y.); 2 Engineering Research Center of Information Network, Ministry of Education, Beijing 100876, China; 3 St. Erik Eye Hospital, Polhemsgatan 50, 112 82 Stockholm, Sweden; wei.zhou@sll.se (W.Z.); leonard.girnita@ki.se (L.G.); stefan.seregard@ki.se (S.S.); 4 Department of Oncology and Pathology, Karolinska Institutet, 171 76 Stockholm, Sweden; 5 Department of Clinical Neuroscience, Karolinska Institutet, 171 76 Stockholm, Sweden; 6 Departments of Ophthalmology and Pathology, Emory University School of Medicine, Atlanta, GA 30322, USA; ophtheg@emory.edu

**Keywords:** BAP1 expression prediction, ophthalmic histopathology images, densely-connected network, deep learning, immunohistochemistry, precision medicine, artificial intelligence

## Abstract

Uveal melanoma is the most common primary intraocular malignancy in adults, with nearly half of all patients eventually developing metastases, which are invariably fatal. Manual assessment of the level of expression of the tumor suppressor BRCA1-associated protein 1 (BAP1) in tumor cell nuclei can identify patients with a high risk of developing metastases, but may suffer from poor reproducibility. In this study, we verified whether artificial intelligence could predict manual assessments of BAP1 expression in 47 enucleated eyes with uveal melanoma, collected from one European and one American referral center. Digitally scanned pathology slides were divided into 8176 patches, each with a size of 256 × 256 pixels. These were in turn divided into a training cohort of 6800 patches and a validation cohort of 1376 patches. A densely-connected classification network based on deep learning was then applied to each patch. This achieved a sensitivity of 97.1%, a specificity of 98.1%, an overall diagnostic accuracy of 97.1%, and an F1-score of 97.8% for the prediction of BAP1 expression in individual high resolution patches, and slightly less with lower resolution. The area under the receiver operating characteristic (ROC) curves of the deep learning model achieved an average of 0.99. On a full tumor level, our network classified all 47 tumors identically with an ophthalmic pathologist. We conclude that this deep learning model provides an accurate and reproducible method for the prediction of BAP1 expression in uveal melanoma.

## 1. Introduction

Uveal melanoma is the most common primary intraocular malignancy among adults. Incidence reaches four to 10 cases per million inhabitants and year, partially depending on geographic location and skin tone [1,2,3,4]. Although less than 4% of patients have detectable metastases at diagnosis, approximately 40% will eventually develop metastases after which the outcome is inevitably fatal [5]. Reliable identification of the group of patients that will develop metastases is therefore key in uveal melanoma prognostication, and a prerequisite for developing an effective treatment to improve outcome.

Several methods have been proposed and implemented, including gene expression assays that show excellent prognostic utility [6]. However, these may not be universally available in clinical routine. Assessments of immunohistochemical expression of one or several biomarkers may offer an alternative. Previous studies showed that expression levels of SPANX-C, ADAM 10, and Raf kinase inhibitor protein are associated with metastatic progression of uveal melanoma [7,8,9]. The BAP1 gene, located on chromosome 3p21.1, encodes a nuclear ubiquitinase involved in the epigenetic modulation of chromatin. BAP1 is one of the most important tumor suppression factors expressed by gene BAP1 [10]. Mutational inactivation of this tumor suppressor is a key event in the acquisition of metastatic competence in uveal melanoma [11].

Earlier studies have shown somatic BAP1 mutations in 45% to 47% of primary tumors and in 81% to 84% of metastatic tumors [12,13,14]. Low nuclear immunohistochemical positivity for BAP1 has been shown to provide significant prognostic information in uveal melanoma and gain equal-level reliability with gene mutation assays [12,13,14,15]. In this situation, the deep learning based image identification method provides a promising prospect [16]. We aimed to establish an affordable, efficient, stable, and easily accessed artificial intelligence alternative to gene mutation assays in the diagnostic evaluation of enucleated eyes.

With the rapid development of artificial intelligence, deep learning based methods have occupied the mainstream in the field of medical image analysis [17]. Early findings have demonstrated that deep learning based artificial intelligence models could capture more abstract and complex features through a self-learning strategy [18,19,20]. Deep learning models have shown significant promotion in pulmonary nodule detection [21], skin cancer classification [22], vascular segmentation [23], and fundus image diagnosis [24]. Moreover, they mine the deep information behind the huge data provided by pathological images, and permit maximizing discoveries [25]. Wang and Khosla [26] used image-patch based deep networks to detect cancer metastasis. Coudray and Moraria [27] employed InceptionV3 [28] for classification and mutation prediction in non-small cell lung cancer histopathology images. In another study [29], Bi and Kim designed stacked fully convolutional networks for cell segmentation of rectal cancer.

Beyond these findings, in our research, we employed a densely-connected neural network [30] for BAP1 expression prediction in ophthalmic histopathology images. In the field of computer vision, densely-connected networks have achieved state-of-the-art in nearly all sub-areas such as image identification [30], image semantic segmentation [31], and object detection [32]. In general, a high-resolution ophthalmic histopathology image usually contains hundreds of millions of pixels and more information than a human observer can process. In our study, we used a patch-based method to settle the resolution issue and cropped the image into unified 256 × 256 patches for the classification of uveal melanoma BAP1 expression. To the best of our knowledge, this is the first time deep learning has been applied to the BAP1 expression prediction task in particular, and to ocular tumor pathology in general.

Our contributions can be summarized as:

- We employed a densely-connected deep classification network for the recognition of nuclear BAP1 expression in immunohistochemical stained eye tissue with uveal melanoma for the first time. Our network has achieved an expert-level performance.
- We created an image dataset that was specialized for use for BAP1 expression in the uveal melanoma.
- We have provided an affordable, efficient, stable and easily accessed approach for uveal melanoma prognostication in a clinical setting.

## 2. Materials and Methods

### 2.1. Dataset Acquisition

Eyes with uveal melanoma that were enucleated at the Ophthalmic Pathology and Oncology Service at St. Erik Eye Hospital in Stockholm between 1979 and 1989 were collected and patients’ medical charts reviewed. Inclusion criteria were (1) histologically proven uveal melanoma, (2) availability of sufficient formalin-fixed paraffin-embedded (FFPE) tissue for immunohistochemical staining and proper representation of tumor histopathology, (3) availability of the following clinicopathological features: gender, age at enucleation, primary tumor location, cell type, according to the modified Callender classification, largest basal diameter (LBD) and tumor thickness, and (4) availability of survival data (time to death or last follow up as well as cause of death). Exclusion criteria were (1) tumor fully necrotic or hemorrhagic, (2) tumor originating in the iris, and (3) prior history of plaque brachytherapy and/or transpupillary thermotherapy (TTT). Seventeen eyes from 17 patients met the inclusion and exclusion criteria.

An American cohort was assembled for further validation. Eyes submitted with a diagnosis of malignant melanoma to the L.F. Montgomery Laboratory, Emory University, Atlanta, USA between 2008 and 2017 were considered. Patients’ medical records were reviewed for clinicopathological data and gene expression classifications (Decision Dx-UM; Castle Biosciences Inc., Phoenix, AZ, USA) [6]. The same inclusion and exclusion criteria were applied and met by 30 eyes from 30 patients. All subjects gave their informed consent for inclusion before they participated in the study.

The study followed the tenets of the Declaration of Helsinki. The protocol for the collection of specimens and data from St. Erik Eye Hospital, Stockholm, Sweden was approved by the regional ethical review board in Stockholm (project identification code 2016/658-31/2), and the protocol for the collection of specimens and data from the Emory Eye Center, Atlanta, USA by the Emory Institutional Review Board (project identification code AM1_IRB00105948). No protected health information was transferred to any parties outside St. Erik Eye Hospital.

### 2.2. Histology

Enucleation specimens were fixed in formalin (10%) and embedded in paraffin; 4 *µ* thick pupiloptic nerve sections that included the center of the melanoma were mounted on one glass slide each. Sections were then deparaffinized with xylene and rehydrated through a graded series of ethanol and distilled water. The sections were stained with hematoxylin eosin and periodic acid Schiff. The slides were evaluated qualitatively and quantitatively with a light microscope (Carl Zeiss AG, Oberkochen, Germany). The LBD, thickness, and cell type (spindle, epitheloid, mixed) of the primary tumor, and any scleral invasion, extrascleral extension, or rupture of Bruch’s membrane were recorded.

### 2.3. Immunohistochemistry

The paraffin blocks were cut into 4 *µ* sections, pretreated in EDTA-buffer at pH 9 for 20 minutes and incubated with mouse monoclonal antibodies against BAP1 at dilution 1:75 (Santa Cruz Biotechnology, Dallas, TX, USA), according to the manufacturer’s instructions, and finally counterstained with hematoxylin and rinsed with deionized water. The deparaffinization, pretreatment, primary staining, secondary staining, and counterstaining steps were run in a Bond III automated IHC/ISH stainer (Leica, Wetzlar, Germany). The dilutions were gradually titrated until optimal staining was achieved, according to manual control.

### 2.4. Annotation and Preprocessing

After sectioning and staining, the Swedish glass slides were digitally scanned at ×400 at the Center of Molecular Medicine, Karolinska University Hospital, Stockholm, Sweden. The American glass slides were scanned at ×200, to allow for validation of our network in a lower resolution, at the Winship Research Pathology Core Laboratory, Winship Cancer Institute of Emory University, Atlanta, USA. Both institutions used a Nano Zoomer 2.0 HT digital scanner (Hamamatsu Photonics K.K., Hamamatsu, Japan). 

**Figure 1:**
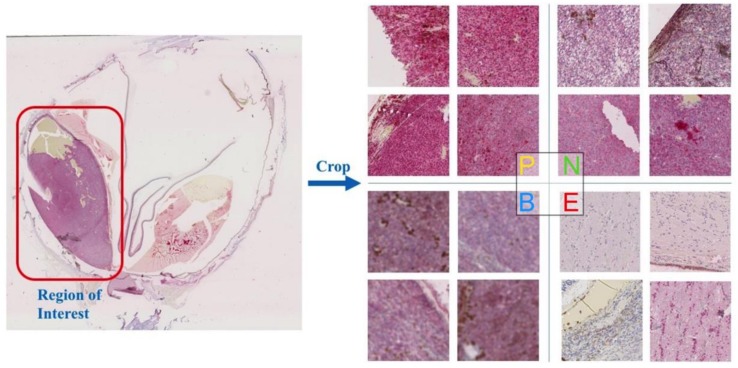
One sample from the dataset of the original scanning of the BAP1 stained uveal melanoma slides and the image patches (256 × 256) cropped from the regions of interest in this image. All patches were divided into four categories: P-positive, N-negative, B-blurred, and E-excluded. We randomly sampled four patches in each category for illustration.

Slides scanned at ×400 and ×200 had a resolution of 227 and 454 nm/pixel, respectively. The regions of interest around the uveal melanoma on each scanning were chosen manually, avoiding areas of tissue or staining artifacts, intense inflammation, fibrosis, necrosis, and poor fixation. The regions of interest were chosen at ×100 and ×50, and were then cropped into multiple 256 × 256 pixel patches to allow for fine detail and reasonable work load. Some patches without tumor tissue were included for the balance of the different categories and robustness of our model. Finally, we obtained a dataset in which there were a total of 8176 histopathology image patches. Some samples of our images are illustrated in Figure 1.

All patches were classified into four categories:

- Positive: Positive BAP 1 patches (retained nuclear expression) (2576 patches).
- Negative: Negative BAP 1 patches (lost nuclear expression) (4720 patches).
- Blurred: Cannot be distinguished/Too vague (560 patches).
- Excluded: Other tissues/Tumor free (320 patches).

**Figure 2:**
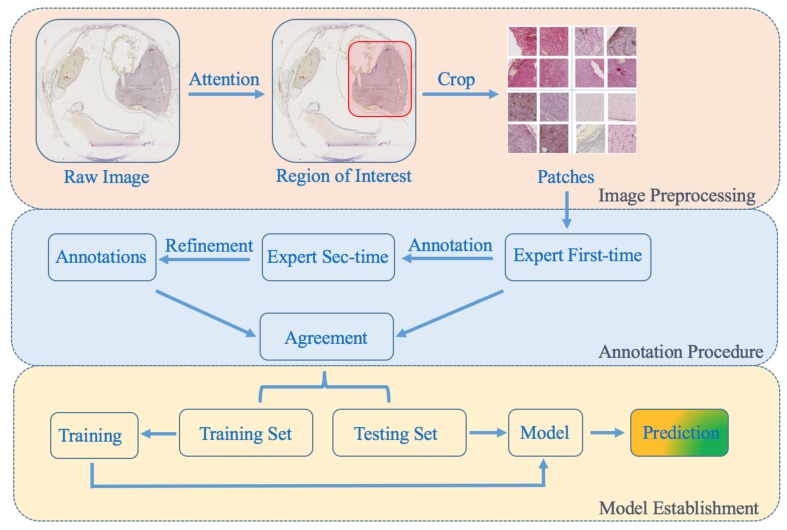
The data flow in our research. First, the raw images were cropped into patches. Second, the patches were finely annotated through two steps by an ophthalmic pathologist. Finally, the dataset was separated into two subsets and fed into the network for training and prediction.

To normalize the standard of diagnosis, each patch was annotated twice by an ophthalmic pathologist (G.S.), first broadly into one of the four categories above-mentioned, then corrected and finely refined. The data collection flow is shown in Figure 2.

In our experiments, we randomly divided the dataset into a training subset and a testing subset. The training set was used to train the model parameters, and the testing set was used to verify the effect of the models. We selected 6800 image patches for training and 1376 for testing. Furthermore, we used the American cohort to test our network in a lower resolution environment. To balance the image intensity variance between different equipment and different staining degrees, we standardized the tumor image to (0, 1), which means that the tumor image has been subtracted by the mean value and divided by the standard deviation of the image intensity. Moreover, basic image augmentation methods such as rotation, shift, rollover, and mirror transformation were randomly used before each forward training procedure.

### 2.5. Manual BAP1 Classification

Nuclear BAP-1 reactivity was assessed by an ophthalmic pathologist (G.S.) using a 4-point scoring system. Briefly, nuclear immunoreactivity was evaluated in approximately 100 cells in each 256 × 256 pixel patch (at ×50–100). The level of nuclear BAP1 expression was classified as low if <33% of the tumor cell nuclei were stained above the background, and as high if *≥*33% were positive [15].

### 2.6. Densely-Connected Deep Classification Network

Deep learning has developed rapidly in computer vision. The deep classification network is a hierarchical neural network, which aims at learning the abstract correlations between the input raw data and the annotations. The mechanism of the deep models is to simulate the mechanism of human visual perception of the eyes and analysis of the brain [30]. Deep models usually consist of layers of convolution, nonlinear rectification, and specific pooling. From a biological perspective, neurophysiological evidence for hierarchical processing in the mammalian visual cortex provides the inspiration for deep networks with cascaded convolutional operations [33]. Rectification nonlinearities are usually introduced into the networks in order to explain their firing rates as a function of the input [34], and pooling is motivated by the function of the cortical complex cells [32,35,36,37].

**Figure 3:**
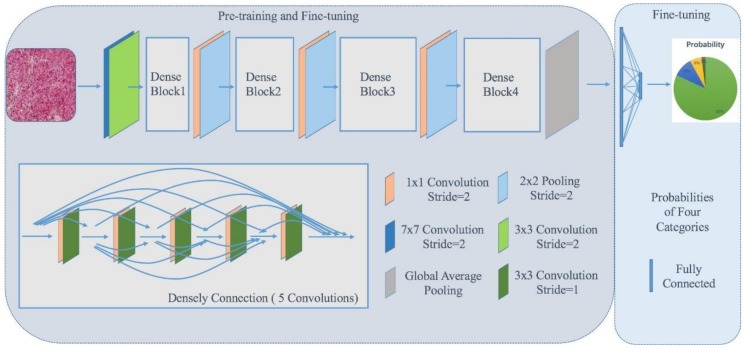
Illustration of our densely-connected deep network. We employed the DenseNet-121 based network in which there are four dense blocks. These four dense blocks are composed by densely-connected cascaded convolutional operations with different groups of convolutions with the number of 6, 12, 24, 16, as shown in the left bottom. The dense connection is shown by five convolutional groups. Each group of convolutions is composed of one 1 × 1 convolutional layer and one 3 × 3 convolutional layer. All of the dense blocks are connected by one 1 × 1 convolutional layer and one 2 × 2 pooling layer.

In this study, we employed a densely-connected deep network similar to DenseNet, which combines the basic convolutional architecture and the dense connection strategy. We used a DenseNet-121 based network with the pre-trained procedure on the dataset of ImageNet [38], and applied the pre-trained parameters before the fully-connected layers. An illustration of our network is shown in Figure 3.

### 2.7. Training Process and Implementation Details

In order to optimize the parameters for finding the optimal mapping relationship between the input images and the labels, we used an iterative process with the backpropagation algorithm, and for backpropagation, we chose the cross-entropy loss function over the categories and used standard stochastic gradient descent (SGD) with weight decay 1 × 10−4. The loss function is shown as follows (formula 1):

(1) $$L\left(w\right)=\frac{1}{N}\overset{N}{\sum_{n=1}}\left[\left(label_{n}\right)log\left(p_{n}\right)+\left(1-label_{n}\right)log\left(1-label_{n}\right)\right]+\lambda \left|w\right|$$

where *w* is the parameter of the model that needs to be trained; *N* is the training sample number; *label_n_* is the true BAP1 expression status; *p_n_* is the predicted probability of BAP1 expression; and *λ* is the regularization term, which has been set as 3 × 10−4 to avoid over-fitting.

We employed an end-to-end training strategy with the batch size = 32. First, we pre-trained our models on the ImageNet dataset. Then, we fine-tuned on our own histopathology dataset for 100 epochs. Inspired by Deeplab [39], we used the “poly” learning rate policy. The learning rate has changed along with the training steps by multiplying (formula 2):

(2) $${\left(1-\frac{iter}{maxiter}\right)}^{power}$$

where *iter* represents the training steps and *maxiter* represents the maximum training iterations. We set the power = 0.95 and initial learning rate as 1 × 10^−3^. All of the matrix calculations were implemented on two NVIDIA Geforce 2080 graphics processing units (GPU) (NVIDIA, Santa Clara, CA, USA). All experiments were implemented by the PyTorch toolkit (Open Source software package, available at http://www.pytorch.org) [40] and Python 3.6 (Python Software Foundation, Wilmington, DE, USA).

### 2.8. Evaluation Metrics

The purpose of the prediction of nuclear BAP1 expression was to classify each patch in the histopathology images into four categories: positive, negative, blurred, and excluded. The two most important categories that require more attention were the positive and negative. By comparing the predicted results of the model with the values of the annotations, we obtained four types of indicators:

- True Positive (TP): the total number of positive pixels correctly predicted;
- False Positive (FP): the total number of negative pixels incorrectly predicted;
- True Negative (TN): the total number of negative pixels correctly predicted; and
- False Negative (FN): the total number of positive pixels incorrectly predicted

Through these four basic indicators, we calculated some basic evaluation metrics such as accuracy, sensitivity/recall, specificity, and the comprehensive evaluation indicator F1-score. Recall, accuracy, and F1-score are three relatively important metrics in medical image analysis. In our experiments, we used nearly all of the metrics above-mentioned. The calculation formulas are as follows (formulas 3–7):

(3) $$Accuracy=\;\frac{TP+TN}{TP+TN+FP+FN}$$

(4) $$Sensitivity/Recall=\;\frac{TP}{TP+FN}$$

(5) $$Specificity=\;\frac{TN}{TN+FP}$$

(6) $$Precision=\;\frac{TP}{TP+FP}$$

(7) $$F1=\;2\;\times \frac{Precision\;\times \;Recall}{Precision+Recall}$$

## 3. Results

### 3.1. Descriptive Statistics

The mean age at enucleation of patients included in this study was 63 years (SD 14). Of the 47 patients, 25 were women and 22 men. Forty-four had tumors that originated in the choroid and three had tumors that originated in the ciliary body. No tumor originated in the iris. The cell type was mixed in 33 patients, spindle in eight, and epitheloid in six. Mean tumor thickness was 9.2 mm (SD 3.2) and mean diameter was 16.1 mm (SD 3.7). Twelve tumors were of gene expression class 2, and 14 of class 1a or 1b. For 21 tumors, gene expression classification was not available. Twenty-two tumors had low nBAP-1 expression and 25 had retained/high expression. Mean follow-up time for patients that did not develop metastases was 89 months (SD 98, Table 1).

### 3.2. Model Performance in the Prediction of BAP1 Expression

**Figure 4:**
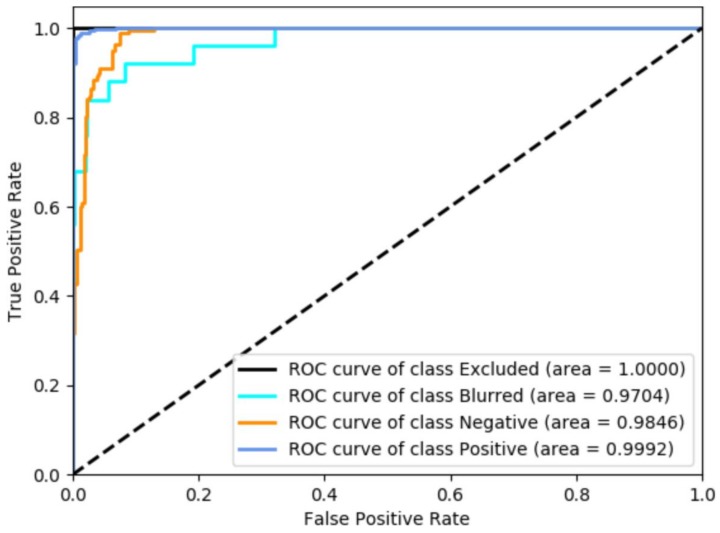
Receiver operating characteristic (ROC) curves of the four categories. Class positive = retained nuclear BAP1 expression. Class negative = lost nuclear BAP1 expression.

In our research, we trained our network on the 6800 image patches and evaluated on the other 1376 patches. As a result, we achieved a sensitivity/recall of 97.09%, a specificity of 98.12%, and an overall diagnostic accuracy of 97.10% in the prediction of nuclear BAP1 expression. The ROC curves are shown in Figure 4.

### 3.3. Model Performance Compared with Other Methods and Human Experts

First, we applied our model on the Swedish cohort and compared it with several different previously established methods with a unified standard: Support vector machine (SVM) [41], VGGNet [42], InceptionV3 [28], and ResNet [18]. SVM is a machine learning method with few-shot learning strategy. Before the upsurge of deep learning, SVM was one of the most widely used methods in data classification. VGGNet and InceptionV3 are two inchoate deep classification networks. ResNet has been a popular method for the rapid progress of image classification in the last three years. We applied each method five times and calculated the average operation. The comparisons of the performance of these methods are listed in Table 2. Our densely-connected network achieved a better performance than the compared methods.

To verify the effectiveness and robustness of our obtained model, we also evaluated our network on the American cohort. Despite the lower image resolution, our network achieved acceptable results, as shown in Table 3.

For the purpose of comparing our network with a human observer, we reinvited the ophthalmic pathologist (G.S.) to diagnose the testing set of the Swedish data. We used our twice labeled annotations as the evaluation standard. Under this unified standard, the metrics gained by the pathologist were calculated. The results are presented in Table 4. Again, the performance of our network was very close to the human observation.

### 3.4. Regression Analysis and Survival

The same threshold for the classification of nuclear BAP1 expression used for individual patches was applied to full tumors, mimicking a previously used method for the classification of BAP1 expression [15]: If <33 % of all tumor cell nuclei in the three most intensively stained patches from a tumor were stained above the background, the tumor’s BAP1 expression was classified as low. If ≥33% were positive, the tumor’s BAP1 expression was classified as high. This yielded identical classification of all 47 tumors by our network and an ophthalmic pathologist. These classifications were then used for survival analyses.

In the multivariate Cox proportional hazards analysis with tumor diameter and BAP1-classification as covariates, neither the tumor diameter (hazard coefficient 170,796.6 for each increased millimeter in diameter, 95% CI 0–3 × 10^132^, *p* = 0.94) nor BAP1-classification (hazard coefficient low versus high expression 1.1, 95% CI 0.9–1.5, *p* = 0.33) were independent predictors of metastasis. Individually, BAP1-classification was a significant predictor (hazard coefficient 26.0, 95% CI 3.3–205.9, *p* = 0.002), but not tumor diameter (hazard coefficient 1.2, 95% CI 0.9–1.6, *p* = 0.12).

**Figure 5:**
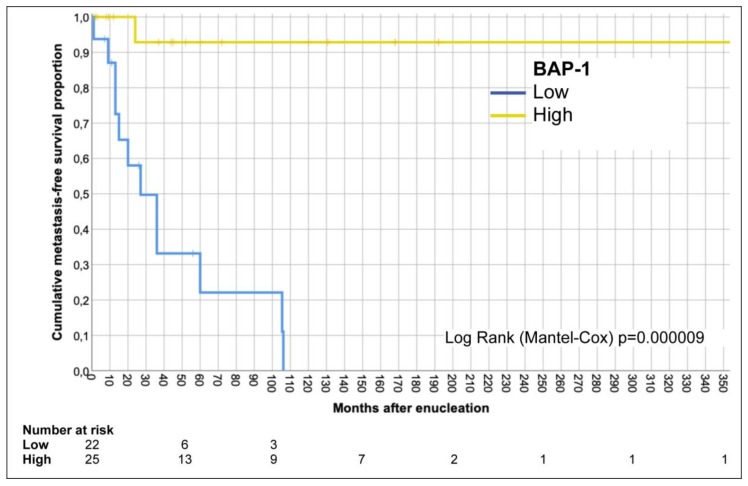
Kaplan-Meier curve. Patients with tumors that had low BAP1-expression, identically classified by both an ophthalmic pathologist and the deep learning network, had significantly shorter cumulative metastasis-free survival than patients with tumors that had high BAP1-expression (Log-Rank *p* = 0.000009).

In the Kaplan-Meier analysis as shown in Figure 5, patients had significantly shorter metastasis-free survival if their tumors had low BAP-1 expression (Log-Rank *p* = 0.000009).

### 3.5. Abstract Perception of Deep Networks

**Figure 6:**
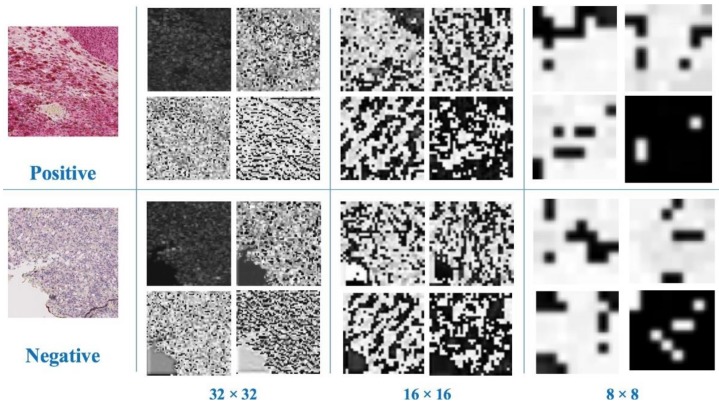
Several samples of convolutional feature maps in our networks with different downsampling strides. We visualized two groups of feature maps from two categories: positive and negative. With the increase of depth, the feature maps became more abstract from detailed enhancement to global perception. For different categories, we sampled the feature maps with the same layers and channels (four channels in each layer). The coding of feature maps from the two categories was distinct.

In the deep learning models, the most effective mechanism for classification is the feature representation ability. By learning from 6800 tumor images, the deep learning model detected features that are strongly associated with nuclear BAP1 expression. Unlike the previous machine learning methods that have focused on handcrafted features, deep learning models can learn the features in an implicit and abstract way. Several convolutional feature maps were visualized at the end of dense block 2, 3, 4 in our densely-connected model, as illustrated in Figure 6. The feature maps were further sampled with channel number 50, 100, 150, and 200 in each layer. We found that the features became more and more abstract, from distinct details to global representation, with the increase of layers. This learning strategy is totally different from the traditional way. With the training of millions of parameters, the features were gradually related to nuclear BAP1 expression. The feature maps of the same convolutional layers were also compared. Our model was capable of learning the specific features of different categories respectively.

### 3.6. Visualization of the Predictive Results

**Figure 7:**
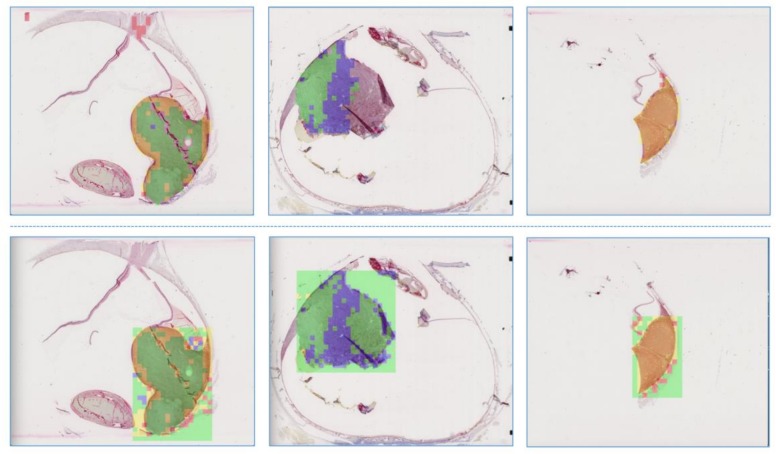
Visualization of the effectiveness of our network. (**Line 1**) Three raw histopathology images with annotations. (**Line 2**) The corresponding predictions in the regions of interest of the three samples. Yellow, green, red, and blue corresponds to positive (retained nuclear BAP1 expression), negative (lost nuclear BAP1 expression), excluded, and blurred, respectively.

**Figure 8:**
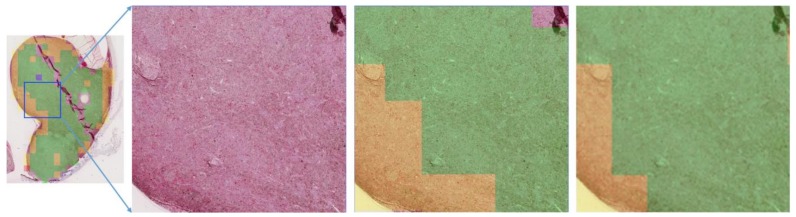
Prediction of BAP1 classification with our network in one detailed area of interest. (**Left**) Overview of the specimen with a “collar button” configuration. (**Middle left**) Original image of the region outlined in the blue box. (**Middle right**) Annotation by ophthalmic pathologist. (**Right**) Prediction by our network. Yellow areas correspond to BAP1-classification “high” and green to “low”.

In order to demonstrate the effectiveness of our deep learning model, we selected another seven annotated uveal melanoma slide scans for prediction and visualization. Three of them are shown in Figure 7. For a more detailed illustration, we amplified one area of interest in the predictions, as shown in Figure 8. 

## 4. Discussion

Our study demonstrated that our densely-connected deep learning model can be used to assist in the diagnostic evaluation of nuclear BAP1 expression in the uveal melanoma from histopathology slides. We trained the deep learning model with 6800 histopathology image patches from clinical slides and validated the performance with another 1376 patches. This model achieved a high sensitivity and specificity for the classification by an ophthalmic pathologist. It suggests that our model could be beneficial for pathologists in the diagnostic procedure, primarily to increase reproducibility. Our study has also demonstrated the superiority of our deep learning model when compared to other traditional image classification methods. Previous studies have used handcrafted clinical factors [43] (such as age, gender, lifestyle, tumor stage) and immunohistochemistry-based/radiomics-based feature engineering methods [44,45,46] to predict gene mutation status. However, these methods could only gain low-level visual features or simple high-level features. Recent studies have proven that using histopathology/pathology images [27] to predict prognosis is a promising and efficient approach to replace genetic testing in the clinic. In the task of nuclear BAP1 expression recognition, clinicians have the challenge of counting the proportion of tumor cells with nuclear stain above the background, which may generate considerable intra- and interobserver variability [47]. However, there are abstract features in the surrounding tissues, which can probably be associated with BAP1 expression [48]. Therefore, it is noteworthy that our network has shown the ability to mine global features that are difficult to formulize, but are essential for detecting nuclear BAP1 expression [49].

To our best knowledge, this is the first study to use the deep learning method to predict the nuclear BAP1 expression status in ophthalmic histopathology images. Furthermore, previous studies that have combined medical image analysis with deep learning have mainly focused on the pathological diagnosis and partially in computer engineering applications such as gastric cancer segmentation in digital pathology images [50], polyp detection in endoscope images [27], and lung cancer detection in computed tomography (CT) scans [51]. In our work, we concentrated more on the combination of bioinformatics research with deep learning based computer vision methods, which is more similar to simple medical research, and not computer engineering. This phenomenon has shown that deep learning is a potential way to improve the accuracy, reliability, and efficiency of the details in medical research. Accelerating the fusion of deep learning methods in the field of pure medical research is imperative (not just the computer-aided diagnostic applications).

Despite the conspicuous performance of the deep learning method, our study also has some limitations. First, the histopathology slides used to train the deep learning network were from two centers, but may not fully represent either the diversity of nuclear BAP1 expression in uveal melanoma, or the spectrum of interlaboratory differences in the results of fixation, sectioning, and staining. More slides from other hospitals would be needed for further testing. The datasets from these two centers were intentionally scanned at different magnifications. The resulting lower resolution of images in the American cohort is the most likely reason for the slightly lower congruence with pathologist classifications. Second, the small number of slides and unbalanced number of four different categories: positive, negative, excluded, and blurred is a limiting factor for model training. Third, we relied on a human expert’s assessments of protein expression to train our network, and not an external objective factor such as sequencing of the *BAP1* gene. This means that we have relied very heavily on the evaluations of one ophthalmic pathologist and his fellow ophthalmologist to develop our network, and that it can outperform humans in terms of reproducibility, but only perfectly imitate and not exceed their performance in interpretation of the level of BAP1 expression. Fourth, as shown by Farquhar et al., loss of BAP1 expression may be seen in a relevant proportion of cases without a *BAP1* mutation [49], and vice versa: there is a possibility that a mutated and non-functional BAP1 is expressed in the nuclei of tumor cells, leading to a false impression of normal protein levels. This means that one cannot make reliable assumptions on the integrity of the gene based on the expression of the protein. Fifth, artificial intelligence and interpretation of deep-learning networks may not be intuitive and may cause distrust in the results of its operation. Exploiting the interpretability of the deep learning method, optimizing its application and utility in the field of medical research is still an imperative task.

In the future, we will concentrate more on the establishment of the dataset to make the images more varied from multiple sources. We will try to explore suitable normalization methods to reduce the differences between different datasets. We plan to extend the recognition from BAP1 expression to other metastasis-related proteins by applying similar deep networks. Furthermore, the correlation between BAP1 expression and other metastasis-related proteins should be explored in future work. We hope that our algorithm will play a role in the prognostication of uveal melanoma, and possibly also find applications for other tumors. Finally, simple engineering of this research might be beneficial for clinicians in practice. 

## 5. Conclusions

In conclusion, we applied a densely-connected deep neural network for the recognition of nuclear BAP1 expression. Our network achieved an excellent performance, which was comparable to that of an ophthalmic pathologist. Our study suggests that artificial intelligence approaches are promising in the analysis of histopathology images and may provide reliable information about prognosis.

## Figures and Tables

**Table 1:** Characteristics of patients and tumors included in this study.

*n* =	47
Mean age at diagnosis, years (SD)	63 (14)
Sex, *n* (%)	
Female	25 (53)
Male	22 (47)
Primary tumor location, *n* (%)	
Choroid	44 (94)
Ciliary body	3 (6)
Iris	0 (0)
Cell type, n (%)	
Spindle	8 (17)
Epithelioid	6 (13)
Mixed	33 (70)
Mean tumor thickness, mm (SD)	9.2 (3.2)
Mean tumor diameter, mm (SD)	16.1 (3.7)
Previous brachytherapy or TTT, *n* (%)	
No	47 (100)
Yes	0 (0)
AJCC T-category, *n* (%)	
1	0 (0)
2	12 (26)
3	24 (51)
4	11 (23)
Gene expression class, *n* (%)	
1a	8 (17)
1b	6 (13)
2	12 (26)
Na	21 (45)
BAP-1 classification, *n* (%)	
High	25 (53)
Low	22 (47)
Follow-up months, mean (SD) *	89 (98)

SD, Standard deviation. TTT, Transpupillary thermotherapy. Na, Not available.

**Table 2:** Comparisons with four other classification methods benchmarked against an ophthalmic pathologist.

Method	Sensitivity	Specificity	Accuracy	F1-Score
SVM	88.76%	91.52%	89.50%	89.20%
VGGNet	91.79%	97.56%	94.87%	94.20%
InceptionV3	95.56%	93.88%	94.27%	94.21%
ResNet-101	95.26%	95.23%	95.25%	95.11%
Our network	97.09%	98.12%	97.10%	97.81%

**Table 3:** Evaluation of our network on the American cohort benchmarked against an ophthalmic pathologist.

Method	Sensitivity	Specificity	Accuracy	F1-Score
Our network	92.09%	93.12%	92.80%	92.96%

**Table 4:** Comparison of classifications, benchmarked against ophthalmic pathologist.

Method	Sensitivity	Specificity	Accuracy	F1-Score
Our network	97.09%	98.12%	97.10%	97.81%
Ophthalmologist	97.25%	97.81%	97.62%	97.76%

## Abbreviations

The following abbreviations are used in this manuscript:

BAP1	BRCA1-associated protein
IHC	Immunohistochemistry
ROC	Receiver Operating Characteristic
GPU	Graphics Processing Unit
SGD	Stochastic Gradient Descent
SVM	Support Vector Machine
VGG	Visual Geometry Group
ResNet	Residual neural network
CAD	Computer-aided diagnostic
CT	Computed Tomography
